# Anti-breast Cancer Agents Derived from Plants

**DOI:** 10.1007/s13659-014-0048-9

**Published:** 2014-12-03

**Authors:** Dmitri O. Levitsky, Valery M. Dembitsky

**Affiliations:** 1 Unité Fonctionalité et Ingénierie des Protéines (UFIP), Faculté des Sciences et des Techniques, Université de Nantes/CNRS, 44322 Nantes Cedex 03, France; 2 Institute of Drug Discovery, P.O. Box 45289, 91451 Jerusalem, Israel

**Keywords:** Plant extracts, Anti-breast cancer, Juice, Tea, Coffee, Wine

## Abstract

Upon emergence of modern anticancer therapy, medical community is divided into two opposite camps, one of them claiming absolute necessity of using isolated or synthesized chemical compounds for efficient patient treatment and another one advocating alternative cancer therapies, in particular those based on natural sources, including extracts from plants. It seems, in reality, that the two camps are reconcilable: while natural sources, plant extracts or juices play both curative and protective role, drugs represent the ultimate possibility to inhibit or reverse tumor development. In this paper we tried to analyze anti-breast cancer potencies of quite a few extracts from different plant sources and to compare their anti-proliferative efficiency of crude extracts with actions of their purified ingredients.

## Introduction

**Fig. 1:**
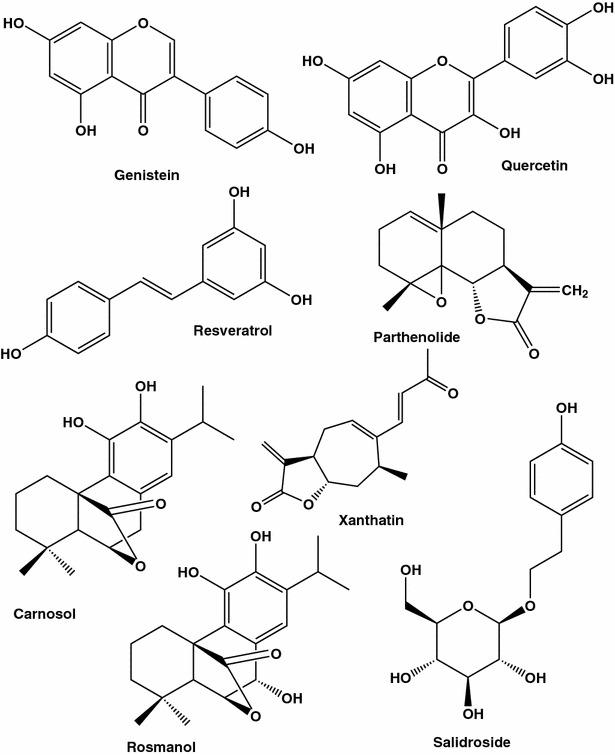
Selected anti-breast cancer agents isolated from different plant species

Breast cancer is the second overall cause of death for women. Most therapeutic drugs (Fig. 1) are derived originally from plants, in particular flowers, fruits, fungi, and/or lichens [1–15].

**Fig. 2:**
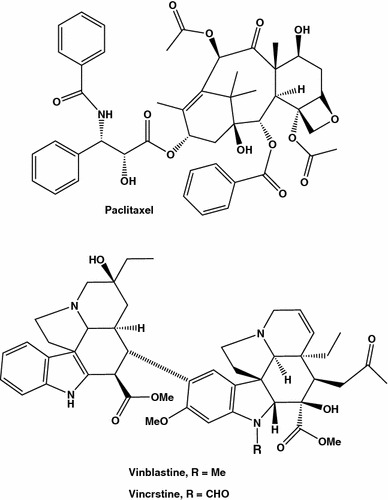
Akaloids possessing highest anti-breast cancer activity

In the 1960s, scientists discovered that an extract from the bark of the Pacific yew tree (*Taxus brevifolia*) could be used to fight cancer. The toxic action of yew tree extracts and even vapors emanating from the tree are known since antiquity (taxon = poison). Its active substance, taxol (Fig. 2), was found to be very efficient in arresting cell cycle by blocking microtubules depolarization, and Taxan™ is used at present as a drug of reference in estimating activity of new substances destined for treatment of breast and ovary cancers [16–19].

Other microtubule inhibitors or “tubulin interactive agents”, alkaloids vincrstine and vinblastine (Fig. 2), isolated from Madagascar periwinkle *Catharanthus roseus* or *Vinca rosea,* were found as well be efficient in breast cancer treatment. The mentioned drugs are ones in hundreds of naturally occurring substances used for centuries to treat the disease and to promote health, and all the time medicine relies mostly on plants, plant extracts, and other plant products. The search for new anticancer agents of natural and synthetic origin rests a very active field of scientific activity, notably due to acquired single- and multi- drug resistance of tumor cells, a relatively new phenomenon appeared due to intense use of anticancer compounds. Thus anticancer drugs which do not serve as substrates for multidrug transporters, such as “breast cancer resistance protein”, BCRP and “multi drug resistance protein”. P-glycoprotein, may considerably increase chemotherapy efficiency [20–23].

## Plant Extracts as Potential Inhibitors of Breast Cancer

**Fig. 3:**
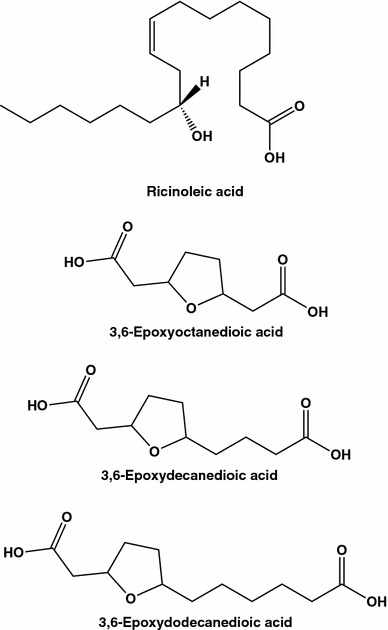
Structures of ricinoleic acid and epoxy dicarboxylic acids

**Fig. 4:**
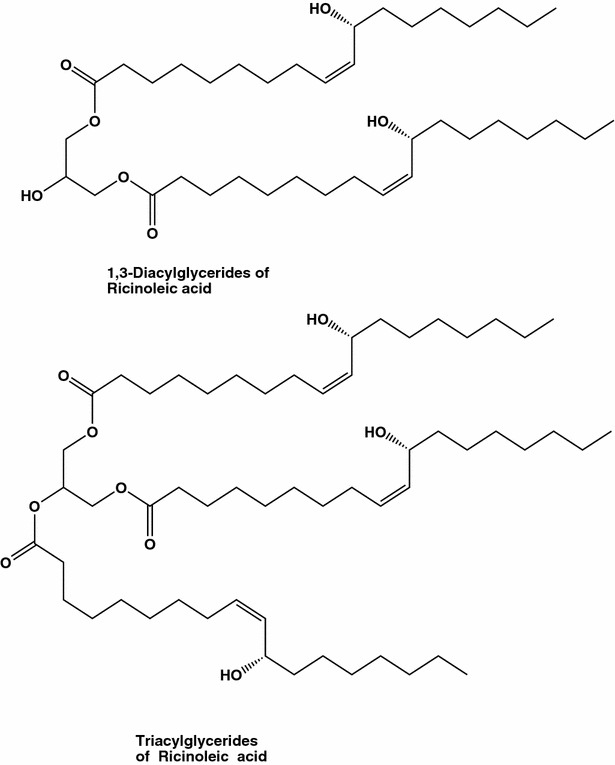
Bioactive di- and triacylglycerides of ricinoleic acid

Ancient Egyptians were the first to mention castor oil as a medicine and since then this oil, also known as Palma Christus, has been used as a folk medicine [15, 24, 25]. Castor oil packs had been highly popularized by Edgar Cayce, “The Sleeping Prophet”, who recommended, in particular to eradicate tumors near the breast surface. The seed oil from the castor bean *Ricinus communis* is very rich in Δ-12-hydroxy-9-octadecenoic acid (ricinoleic acid, about 90 %, Fig. 3), and contains as minor components phenolic compounds, such as *p*-coumaric acid, ferulic acid, *o*-coumaric acids, syringic, cinnamic, chlorogenic, neochlorogenic, and gallic acids [26–29]. Quite a few of triglycerides of *R. communis* containing ricinoleic acid, have as major components triricinolein (Fig. 4) and, in addition, diricinoleo-triglycerides with ricinoleic acid at the 1- and 3-positions (Fig. 3) [30]. Castor oil is hydrolyzed in the small intestine by pancreatic enzymes, which results in the release of glycerol and ricinoleic acid, although 3,6-epoxyoctanedioic acid, 3,6-epoxydecanedioic acid, and 3,6-epoxydodecanedioic acid (Fig. 4) also appear metabolites. Castor oil and ricinoleic acid easily penetrate deep into the skin and they enhance the trans-dermal penetration of other chemicals.

According to estimates of the World Health Organization, 80 % of the world population, especially those living in Asia, Latin America and Africa, still relies on herbal medicine [31–36]. No wonder that screening of natural sources for different kinds of biological activity and medicinal potency are performed mostly on indigenous, often exotic plants. Quite often, an anti-proliferative activity of the plant extracts revealed in studies in vitro was not associated with the medicinal effects of herbal formulations used by local herbalists.

*Vernonia amygdalina* (VA), is an edible African mountain plant of the Asteraceae family. It is known as “bitter leaf”, eventually due to high amounts of alkaloids, saponins and tannins [37]. The leaves and the roots of this shrub are used in sub-Saharan Africa for many purposes, e.g. as a tonic, but also to improve digestion, to reduce fever, and to protect organism from intestinal parasites and dermatomes. *V. amygdalina* extracts was also found to inhibit proliferation of breast cancer MCF-7 cells [37]. Extraction of VA with multiple solvents of various polarity indexes yielded three fractions (A1-2, B-3) that significantly inhibited cell growth (*P* < 0.05) at 0.1 mg/mL. At a higher concentration (1 mg/mL), six fractions extracted by hexane, chloroform, butanol, and ethyl acetate inhibited DNA synthesis by 76–98 %. Obtained fractions inhibited also the growth of MKL-F breast cancer cell line [30, 37]. Interpretation of these effects is not simple since *V. amygdalina* leaf extracts contain quite a few potentially anti-cancer active ingredients of different functional and structural properties, such as antioxidants (flavonoids), lipophilic terpenoids (sesquiterpene lactone), and amphiphilic saponins.

**Fig. 5:**
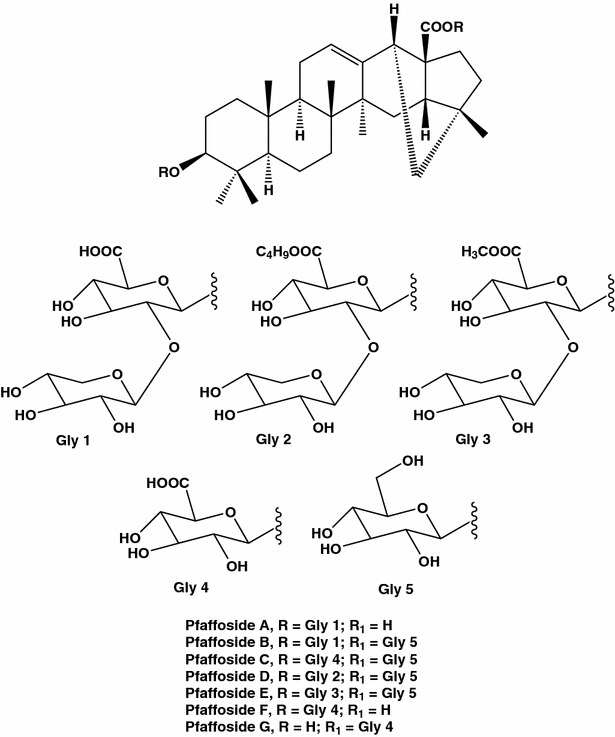
Bioactive saponins pfaffosides A–G isolated from an Amazon shrub Suma, *Pfaffia paniculata*

An Amazon shrub Suma (*Pfaffia paniculata*), also nicknamed Brazilian ginseng and “para tudo” (“for everything”), is used in folk medicine as tonic and assures resistance to stress. On the other hand, extracts from Suma bark or roots were found to possess cytotoxic activity. *P. paniculata* contains in particular pfaffic acid, and saponins pfaffosides A–G (Fig. 5). Toxicological properties of saponins present in different edible plants are wildly recognized. It is supposed that these surface-active glycosidic compounds could be in part responsible for well documented anticancer actions of roots of Suma [38–40]. A mixture of compounds obtained after evaporation of butanolic extract from Suma roots were found to be cytotoxic against MCF-7 cells, starting from the concentration of 0.4 mg/mL [41]. Though damaging of cell and mitochondrial membranes and nuclear structure was observed, detailed mechanisms of action of the extracts on the cancer cells remain to be investigated.

In some screening studies, the portion of potentially useful plants was found to be extraordinary high. Thus 32 plants were collected in the rain Malaysian forests and 143 crude extracts were evaluated for their anti-proliferative activities on two breast cancer cell lines, i.e. MCF-7, the most commonly used model of estrogen-positive breast cancer line, and T47D cells. After performing sulforhodamine-B assay, 13 crude extracts from 11 plant species were found to possess an anti-proliferative activity (IC_50_ for dried extracts was 0.1 mg/mL or lower) [42].

One has to realise that chances to identify really efficient anti-cancer plant extracts are quite low. Indeed, how many plants can be considered as potential sources of active compounds? What is a dose value corresponding to a borderline between extract potency and inefficiency? Two studies mentioned below partially clarify the incertitude.

Brazilian rain forests are characterized by the exceptional variety of the forms of plant species. No wonder that periodically attempts have been made to perform the global screening of extracts from the plants in search of potentially active compounds. Thus more than 1000 aqueous and organic extracts of 351 species were checked at a dose of 0.1 mg/mL for their capacities to suppress growth of MCF7 cells. Surprisingly, only 11 extracts revealed marked cytotoxicity at this the relatively high dose [43].

Another pharaoh study was undertaken in 8-year screening of South African plants [44]. Of a total of 7500 plant extracts screened on 60 human cell lines, the most active were further tested on MCF7, renal cancer and melanoma cell lines. The extracts were separated into 4 groups, according to TGI (total growth inhibition) values: >50 μg/mL (inactive), 15–50 μg/mL (weak), 6.25–15 μg/mL (moderate) and <6.25 μg/mL (potent). Of total of fifty active extracts neither was found to be potent in MCF7 test, and only twenty presented moderate inhibiting activity. At first sight, the conclusions from these two screening studies seem to be frustrating. Nevertheless a labor-consuming and often altruistic approach dealing with the testing of crude plant extracts quite often leads to identification of active compounds and revealing cellular mechanisms involved in the cytotoxic actions [44].

**Fig. 6:**
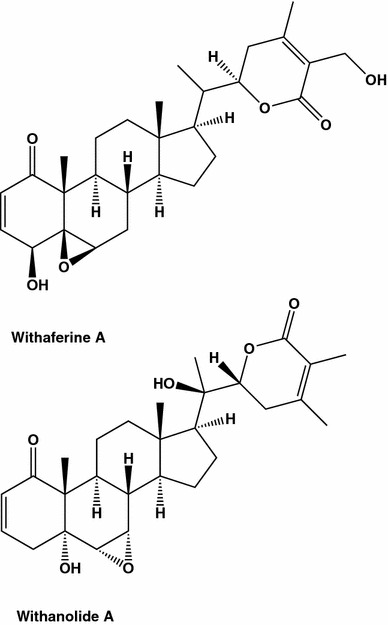
A few bioactive C28-steroidal lactone triterpenoids, withanolides were identified from Indian ginseng (*Withania somnifera*)

**Fig. 7:**
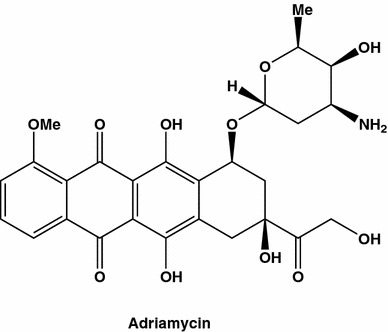
Adriamycin also known as adriacin, adriblastin or adriblastina is an antitumor antibiotic was isolated from *Actinomadura roseola*

*Withania somnifera* (Indian ginseng) is a popular shrub used in Asian traditional medicine, having a 3000 years history. The organic extracts from *W. somnifera* (Solanaceae) were tested among two dozens selected plant species, used in Palestinian traditional medicine [45, 46]. The IC_50_ values obtained after 24 h treatment of cell cultures with the extracts were 150 and 60 µg/mL on for murine fibrosarcoma cell line L929sA, known for its sensitivity to TNF, and MCF7, an ER positive control cell line, respectively. At least two cytotoxic pathways seem to be activated by the *W. somnifera* extract. Among the active constituents in *W*. *somnifera* leaves and roots quite a few C28-steroidal lactone triterpenoids, withanolides (Fig. 6), were identified [47]. Thirteen withanolides were evaluated using antioxidant and cyclooxygenase enzymes inhibitory bioassay-guided test performed on four different cell lines, including MCF7. Three of the withanolides were found to be as efficient as adriamycin (Fig. 7), giving IC_50_ values between 0.23 to 0.40 μg/mL. Withaferine A inhibited growth of breast and colon cancer cell lines event more efficiently than this anticancer drug of reference.

## Effects of Diet Flavonoids on Breast Cancer Initiation and Progression

There are numerous reports that a diet rich in flavonoids may be protective against various types of cancer, from ovarian cancer to lung and pancreas cancers. Nearly 5000 flavonoids were identified in fruits and vegetables. Flavonoids of reference genistein (principle isoflavonoid of soja) and quercetin (present at large amounts in the skins of apples and red onions) have been shown to inhibit tumor cell growth. Though animal and cell culture studies indicate that tumor-preventive effects of electron donor flavonoids are due to their free radical scavenging properties [48, 49], concrete molecular mechanisms for anticancer activity of each active flavonoid are still to be revealed. The spectrum of flavonoid effects spreads from inhibition of cyclin-dependent kinases (CDKs), which are critical for cell growth and division, to blocking angiogenesis and modulating MDR1 activity. For instance, genistein (Fig. 1) was found to be very efficient in inducing apoptosis in MCF-7 and T47D breast cancer cell lines [50]. It is to mention that large-scale clinical trials did not provide decisive conclusions that these antioxidants prevent cancer or slow down the disease. The whole area dealing with health benefits of antioxidants and in particular their anticancer activity enters in turbulence. Indeed, free radical scavengers would protect DNA from damages and prevent cell transformation into malignant type, but on the other hand they may improve cell survival. This double-face nature of antioxidants has been revealed in recent studies showing protective influence of antioxidants on tumor cells. In particular, in extracellular matrix-deprived human breast epithelial cells, antioxidants stimulated fatty acid oxidation, and thus restored ATP synthesis and prevented non-apoptotic cell death [51].

**Fig. 8:**
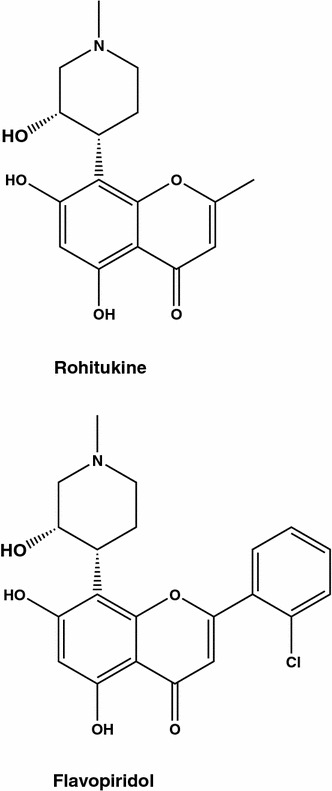
Two anti-breast cancer alkaloids: rohitukine was isolated from *Amoora rohituka*, flavopiridol is a semisynthetic flavone derivative

Among most extensively investigated flavonoids, flavopiridol, catechins, genistein and quercetin are known to prevent cancer and possess anti-tumour activities. Flavopiridol (Fig. 8), also known as alvocidib, HMR 1275 and/or L 86-8275 is a semisynthetic flavone derivative of the natural anti-inflammatory and immuno-modulatory alkaloid rohitukine [52]. Rohitukine (Fig. 8) was isolated for the first time from *Amoora rohituka* (family Meliaceae) in 19 [53, 79] and later from *Schumanniophyton magnificum* [54], and *Dysoxylum binectariferum* [55]. Rohitukine showed moderate cytotoxicity against human HL-60 promyelocytic leukemia and HCT-116 colon cancer cells [56]. After replacement of a methyl group by the chlorophenyl moiety at position 2 of rohitukine, additional pharmacological properties were acquired: [57, 58] from inhibition of different CDKs, thus inducing apoptosis, to modulation of the immune response. In the breast carcinoma MCF-7 cell line, flavopiridol produced arrest of cell cycle in G1, and this action was not dependent on functional p53 [59]. It is to mention that flavopiridol was the first CDK inhibitor to be tested in clinical trials [60].

Another example of a polyphenol which exerts both cell protecting and cancer cell killing effects is quercetin (Fig. 1). This flavonoid present in high amounts in some fruits and vegetables is suggested to exert these opposing actions by playing a role of an antioxidant due to metal chelating and scavenging free radicals, and a role of pro-oxidant due to its ability to generate ROS. The latter species may a priori induce DNA damage [61]. Numerous effects of quercetin on breast cancer cell functions were reported, from inducing p21 (CDK inhibitor) and arrest of cell cycle in G_1_ or G_2_/M [62] to apoptosis which could be due to caspase activation, microtubules modification and an increase of stress proteins expression [61]. No studies were done on tissue level. Concerning results obtained in vitro, in most studies performed on breast cancer cell lines this flavonoid was tested at high concentrations, ranging from 10 to 200 µM. For example, at a relatively high concentration (10 µM) quercetin induced only mild DNA damage, and 94 % of SK-Br3 cells were still viable even after 4-day incubation at this quercetin concentration [61].

To evaluate potential anticancer activities of dietary flavonoids, such as quercetin, one has to compare plasma levels of the ingested natural compound with those used in studies on cancer models. Though only 0.35–1.4 % quercetin ingested at breakfast is excreted in 24 h, its peak level in blood plasma rests quite low: 0.6 µM (apple consumption) and 0.74 µM (onion consumption) [63]. Taking into consideration that consumption of a single onion portion would correspond to intake of about 200 µmol quercetin, one may conclude that a major fraction of the flavonoid is either metabolized or absorbed by the tissues.

**Fig. 9:**
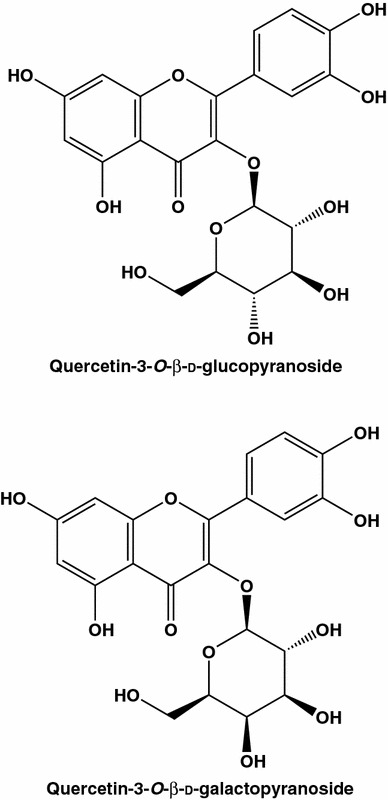
Major flavonoids from apple peels are known as anticancer agents

Apple consumption has been documented to decrease risk a number of chronic diseases, from coronary heart disease to cancer. It is to mention that constituents of apple flesh and apple peel differ considerably. A bioactivity-guided fractionation of Red Delicious apple peels allowed to isolate and characterized twenty-nine compounds, including triterpenoids, flavonoids, organic acids and plant sterols [17]. The major flavonoids in apple peels were found to be quercetin-3-*O*-β-d-glucopyranoside (82 %, Fig. 9) and quercetin-3-*O*-β-d-galactopyranoside (17.1 %, Fig. 9) but, surprisingly (structure see on Fig. 1), not quercetin (0.2 %). When tested on cancer cell lines quercetin and quercetin-3-*O*-β-d-glucopyranoside inhibited proliferation of HepG2 and MCF-7 cells with EC_50_ values of 41 and 49 µM for HepG2 cells and 137 and 24 µM for MCF-7 cells, respectively. Concerning possible antioxidant impact of the isolated phenolic compounds, antioxidant activities of caffeic acid, quercetin, and quercetin-3-*O*-β-d-arabinofuranoside (EC_50_ values <10 µM) were comparable with that of vitamin C.

Attempts were made to correlate anti-cancer efficiencies of apple extracts with those of individual compounds. One of the studies [64] indicates that apple extract and quercetin-3-β-d-glucoside (Q3G) possess anti-proliferative activity against MCF-7 cells (the corresponding EC_50_ values being 71 µg/mL and 46 µM). Two-way combination of apple extracts plus Q3G significantly increased anti-proliferative action on the cells. The EC_50_ values were reduced to 33.8 µg/mL and 10.8 μM, respectively. One has to emphasize that the comparison of efficiencies of crude extracts and their individual ingredients is possible only on wt/volume bases. In this case the latter study would indicate that the apple extract possesses higher anti-proliferative than Q3G. Indeed using wt/volume EC_50_ values (71 and 29 µg/mL, respectively) and taking into consideration very low levels of Q3G in apples [64], one may conclude that the latter quercetin analog should be excluded from the list of potential anti-cancer drugs. Unique synergetic influences of the compounds existing in edible, plants reinforce position of the followers of alternative medicine.

At present, we lack a straightforward evidence of the link between anti-proliferative efficiency of an edible plant ingredient in vitro and its anticancer activity in vivo. For example, Mediterranean diet, characterized by high consumption of fruits and vegetables, is traditionally considered to be one of the healthiest, providing organism protection against cardiovascular diseases and cancer. Numerous studies indicate that extracts from edible plants from Mediterranean areas possess anti-proliferative activity when evaluated on different cancer cell lines. Using the sulforodamine B assay, extracts from sixteen plants, eaten boiled, fried or fresh in Southern Italy, were tested on four human cancer cell lines, including MCF-7 [65]. No clear correlation was obtained between the level of flavonoids and the inhibitory effects. Thus Sow thistle *Sonchus oleraceus* with the highest flavonoid content (33 mg/g of dried extract) was found to be less efficient (8.4 % inhibition at 100 μg of hydroalcoholic extract/mL) than Wild artichoke *Cynara cardunculus* (9 mg/g and 22.7 %) or caper *Capparis sicula* (2.52 mg/g and 42.67 %), very rich in quercetin. On the other hand, extracts from water mint *Mentha aquatica* showing the best antiproliferative activity on MCF-7 cell line (44.7 % inhibition) were characterized by a relatively high flavonoid levels (15.75 mg/mL) and an extraordinary high total phenolic content (337 mg/g).

Another example of a double-face nature of quercetin and the whole class of flavonoids is associated with their structural resemblance to cell estrogens. Estrogen receptors are one of the targets in anti-breast cancer therapy. Flavonoids from medicinal plants are considered as weak phytoestrogens to able to mimic the effects of estrogen. High phytoestrogen consumption is a priori beneficial for postmenopausal women though no straightforward data is yet obtained on their relieving actions on menopausal symptoms. The review of epidemiological data as well as in vitro experiments on ER-positive and ER-negative breast cell lines [66] allowed to postulate that phytoestrogens may exert two opposite actions, depending on their level in blood and concentrations used in experimental studies: At concentrations, not exceeding 10 µM, some phytoestrogens, like genistein, stimulate growth of ER-positive MCF-7 and T-47D cell lines, but not the ER-negative MDA-MB 231/435 breast cancer cell lines. At higher concentrations survival of both types of breast cancer cells of both types decreases. At low doses the phytoestrogens, as ligands of ER, are likely to stimulate directly metabolic pathways providing and/or improving cell proliferation, in particular progression of cell cycle and apoptose inhibition. At higher concentrations, mechanisms which are not dependent on ER signaling, including those associated with prooxidant properties of the flavonoids seem to be triggered. Overall, taking into consideration potential increase of the risk of tumor progession in the presence of circulating RE ligands, decreasing consumption of phytoestrogens as supplements, or those present at high amounts in soy, would be rather beneficial for quite a large women’ population [66].

## Juices as Sources of Potential Inhibitors of Breast Cancer

Phenolic compounds and their glycosides are constituents of many fruits and vegetables, and they have attracted a great deal of public and scientific interest because of their potential anti-carcinogenic and other health-promoting effects as antioxidants [2, 3, 5, 67, 68].

The benefits of a plant, probably most often mentioned in literature, olive tree, for health are known from Bible times. However, in most cases beneficial effects olive oil are emphasized taking in mind high contents of monounsaturated fatty acids, such as oleic acid (comprising 50–80 % of total FA) in the fruits. Since the olives are also known to be rich in polyphenolic antioxidants, preventive anti-cancer properties of olive oil described in medical literature are most likely due to DNA protection from reactive oxygen species (ROS).

Two Spanish groups investigated anti-cancer potencies of extra virgin olive oil (EVOO). In one of the studies [69] effects of dietary EVOO on experimentally induced rat mammary adenocarcinomas were compared with those obtained on animals placed on corn oil diet. Tumors from rats fed the olive oil diet were characterized by a more benign phenotype, while those from rats on corn diet were found to be more aggressive. Moreover the olive oil diet decreased activation of proto-oncogene p21Ras and upregulated the Raf/Erk pathway, compared with the control, whereas the corn oil diet did not modify Ras activity and enhanced the Raf/Erk pathway. It was concluded that two types of oil diets exerted their effects through a different proliferation/apoptosis balance and probably distinct levels of DNA damage.

**Fig. 10:**
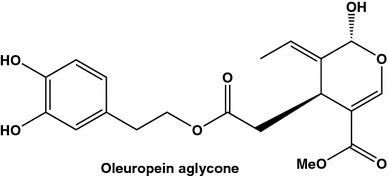
Anticancer agent oleuropein aglycone isolated from virgin olive oil

The olive oil contains dozens of polyphenol, including a strong polyphenolic antioxidant oleuropein and its hydrolyzed derivatives. Anticancer efficiencies of the quantitatively minor phenolic components of EVOO polyphenols, traditionally overshadowed by oleic acid, were tested on MCF-7 (HER2-negative), MCF-7/HER2 and HER2 gene-amplified SK-Br3 breast cancer cell lines [70]. The cell cultures were incubated in the presence of tyrosol, hydroxytyrosol, oleuropein glycoside, and oleuropein aglycone (Fig. 10) at concentration ranging from 6 to 100 µM. The first three polyphenols did not change or modified only slightly viability of the cells, and only oleuropein aglycone produced effects at doze-dependent manners, with IC_50_ values about 50 µM for MCF-7/HER2 and SK-Br3 cell lines. When tested in Cell Death ELISA test, oleuropein aglycone at 25 µM increased apoptosic cell death by factor 1.5 (MCF-5), 2.5 (MCF-7/HER), and 4 (SK-Br3). The authors suggested that an extraordinary anti-oncogenic efficiency of olive oil may be due to actions of both phenolic and fatty acid components. In fact, olive oil is characterized by a unique fatty acid composition (very high level of oleic acid and a low ratio (ω−6)/(ω−3) polyunsaturated FA), which in the combination with oleuropein aglycone provide marked down-regulation of a proto-oncogenic HER2.

 American cranberry (*Vaccinium macrocarpon*) which is very rich in phenolic compounds such as flavonoids, anthocyanins, proanthocyanidins and small phenolic acids has one of the highest free-radical scavenging capacity. It has been shown that 20 h incubation of human breast cancer cells (MCF-7, MDA-MB-231 and MDA-MB-435 lines) in 6.7 % cranberry juice induced death of 20 % of cell population [71, 72].

The genus *Citrus* includes several species, from sweet orange (*S. sinensis*) to mandarine (*C. reticulate*) as well as and multiple hybrids, represents an extraordinary source of flavonoids, and their content in the fruit juices is as high as 1 µg/mL [73]. In previous years most attention has been attracted to citrus flavonoids due to their anti-inflammatory effects and their ability to decrease capillary permeability. Recent studies are focused as well on preventing cancer development by the fruit juices, fruit extracts and individual compounds isolated from the citrus. The major citrus flavonoids include quercitrin (quercetin-3-*O*-rhamnoside), rutin (quercetin-3-*O*-rutinoside), tangeritin, and hesperidin.

**Fig. 11:**
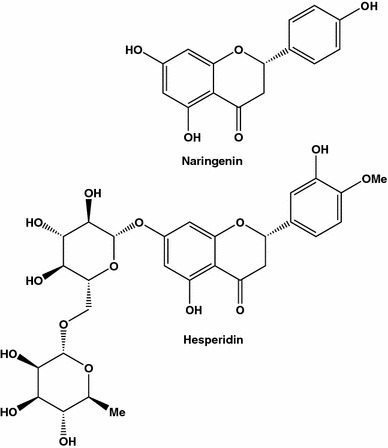
Two anti-breast cancer (MDA-MB-435) compounds: naringenin also known as 4′,5,7-trihydroxy-flavanone, *S*-dihydrogenistein, salipurol, or salipurpol, and second - hesperidin also known as 7-(6-*O*-α-l-rhamnosyl-d-glucoside), atripliside B, cirantin or hesperetin 7-rhamnoglucoside, isolated from grapefruit and orange, respectively

Anticancer actions of hesperidine and naringenin (Fig. 11) were compared with those of other flavonoids of noncitrus origin, including quercetin [74]. When studied on a human breast cancer cell line MDA-MB-435, the tested flavonoids inhibited the cell proliferation at doses varying from 6 (noncitrus baicalein) to 140 µg/mL. Synergistic actions of one-to-one combinations of quercetin with hesperetin and naringenin (with ID values decreasing to 4.7 µg/mL) and naringenin plus hesperetin were also demonstrated.

The discovered synergism may be due to different inhibiting pathways activated by the studied compounds. In another study, inhibiting effects of double strength orange and grapefruit juices on DMBA-induced mammary rat tumorogenesis were compared [75]. In spite of the fact that naringenin and hesperetin are present in oranges and grapefruits at similar concentrations, the juice from oranges was more effective than grapefruit juice. The results of these two studies confirm widely spread, rather intuitive than scientifically justified, belief that active compounds are more effective when consumed from crude extracts than in isolated forms (for update concerning numerous effects of citrus flavonoids and possible mechanisms of their action, see review [76]).

Beliefs in miraculous folk medicine may complicate sober appreciation effects of crude extracts. Thus a traditional Tahitian medicinal plant *Morinda citrifolia* (noni) is believed to produce numerous therapeutic effects, from hypotensive and anti-inflamatory actions to antitumor activity. This plant has also been reported to have antibacterial, antiviral, antifungal, anti-helmint and analgesic activities. Though active compounds of the plant had not been identified, essays have been performed to test its anticancer efficiency. Thus methanol extracts obtained from the fruits of noni were verified for cytotoxic activity using different cancer cell lines. At concentration of 0.1 mg/mL, the crude extract demonstrated very little cytotoxicity to BHK, Hep2, and Vero cells while its cytotoxic activity was found to be pronounced in experiments on neuroblastoma (LAN5) and MCF-7 cell lines [77]. Fruit juice from *M. citrifolia* was shown to treat breast cancer as well to inhibit the metastases and even to destroy metastasized breast cancer cells, see corresponding patent [78].

**Fig. 12:**
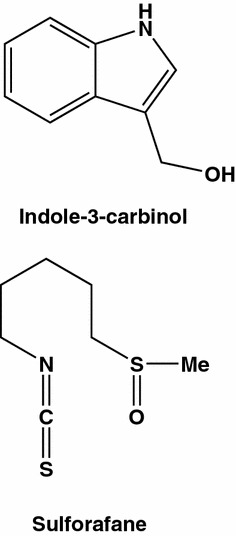
Main compounds of juice from *Brassica olearacea*

The choice between juice (extracts) from the plants and individual compounds as means to realize effective antitumor therapy is painful, especially in case of major women’s killer, a rapidly developing breast cancer. It has to emphasize, however that numerous data have been accumulated during hundreds of years indicating great efficiency of preventive measures, including “anticancer” diets and crude preparations from a large variety of vegetables. An example is the genus *Brassica* (including wild cabbage, cauliflower, Brussels sprouts and broccoli) [79]. Some reports indicate a breast cancer specific action of juices from this plant. Mechanism of antitumor effects of the juices from varieties of *Brassica* is not completely understood, though it is attributed mainly to actions of indole compounds (mainly indole-3-carbinol and sulforaphane, Fig. 12) which induce expression of detoxification enzymes and possess antioxidant activity. In a study performed on MCF-7 (ER+) and MDA-MB-231 (ER−) breast cancer cell lines, the cells were pretreated with increasing concentrations of crude *Brassica olearacea* juice for 72 h which was followed by pulses of [^3^H]thymidine and determination of its incorporation into DNA [80]. The effects on DNA synthesis were dose dependent, and 50 % inhibition was observed at 5–30 mL/L. The IC_50_ for MCF-7 cells was 5 mL/L (with a nearly complete inhibition at 30 mL/L, whereas for other cell lines (Vero, Hep2, ECV30) IC_50_ values were as high as 40 mL/L. It seems that both apoptosis and necrotic pathway are activated in breast cancer cells in the presence of active compounds from the cabbage juices and these pathways do not require expression of estrogen receptors.

Another study indicates that compounds present in cruciferous vegetables may produce opposite effects on estrogen positive cells. Ethyl acetate extracts from cabbage and Brussels sprouts were analyzed for their estrogenic and anti-estrogenic activity by following estradiol-induced proliferation of MCF-7 cells and changes in the level of estrogen-responsive pS2 mRNA. At low doses (5–25 ng/mL) the extracts inhibited the proliferation and decreased pS2 gene expression. However higher concentrations (50 ng/mL–25 µg/mL) increased MCF-7 proliferation [81]. It has been concluded that though both estrogenic and anti-estrogenic compounds seem to be present in cruciferous vegetables, it is not likely that concentrations stimulating estrogen-dependent breast cancer growth could be attained in organism.

## Anti-cancer Properties of Teas and Coffee

Epidemiologic evidence of preventive and curative potential of tea (*Camellia sinensis*) is not consistent. This may be explained by large varieties of tea consumed in Western countries (predominantly black tea) and in Asian countries (green tea). Meanwhile, anticancer properties of green tea are known for many years, and they are presumably due to high content of water-extractable polyphenols which is 5–10 times lower in an oxidized green tea derivative obtained after fermentation (black tea) [82].

**Fig. 13:**
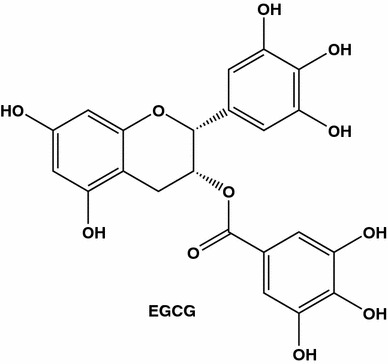
EGCG (also known as epigallocatechin gallate, epigallocatechin-3-monogallate, l-epigallocatechin gallate, epi-gallocatechin 3-*O*-gallate or epi-gallocatechin gallate) is the main green tea polyphenol

Mechanisms underlying these green tea’s properties may include arrest of cell cycle in G1, increase of apoptosis, antioxidant and anti-estrogenic actions. Green tea extracts were shown to have synergetic action with a conventional anti-estrogens drug, tamoxifen. When tested on estrogen receptor-positive MCF-7, ZR75, T47D human breast cancer cells combinations of tea extracts and the drug were found to be more effective in suppressing the cell proliferation than either agent given alone [83]. These data are of potential clinical value since relative effects of the tea extracts were the most pronounced at law doses of tamoxifen, which a priori allows to make more drug therapy harmless and more efficient. Here, it is to mention that green tea flavonoid epigallocatechin gallate (EGCG, Fig. 13) inhibits P-glycoprotein, and the EGCG effects were found to exceed those of quercetin (Fig. 1) and verapamil (Fig. 2) [84].

Direct pro-apoptotic effects of green tea extracts and tea catechins on tumor cells in vitro and in vivo were also demonstrated. When tested on different breast cancer cell lines, EGCG revealed a modest pro-apoptotic activity. However, treatment of 4T1 cells with EGCG in combination with taxol led to a dramatic increase in cell apoptosis compared to treatment with taxol alone [85]. The chemical structure of EGCG provides its ability to be a metal chelating agent, and to possess, depending on its concentration, either pro- or antioxidant activity. Different actions of EGCG were summarized in a review [86]. Other studies also indicate that green tea inhibits angiogenesis. It was demonstrated that crude green tea extracts and EGCG, the main green tea polyphenol, decreased in a dose-dependent manner transcription of vascular endothelial growth factor (VEGF) and inhibited MDA-MB231 breast cancer cell and human umbilical vein endothelial cell (HUVEC) proliferation. In mouse tumor models, green tea extracts suppress angiogenesis which was manifested by decreased necrosis areas and lower blood vessel density in the treated xenografts [83].

In a 7-year study on 472 patients with I-III stages of breast cancer it was shown that increased consumption of green tea, 4–5 cups per day, was associated with decreased numbers of axillary lymph node metastases in the group of premenopausal patients with stages I and II and an increased expression of progesterone and estrogen receptors among postmenopausal ones. It was significantly associated with improved prognosis of stage I and II breast cancer, while no improvement in prognosis was observed in stage III breast cancer [87]. In a long term population-based, case–control study [88], breast cancer risk was related with initiation of green tea consumption and menopausal status. In the group of premenopausal women, a direct relationship was found between the reduced risk and years of green tea drinking as well as amount of monthly consumed tea. Surprisingly, among postmenopausal women decreased risk of breast cancer was associated with an older age of initiation of the tea drinking. Globally, this study indicated that regular green tea consumption was weakly inversely associated with breast cancer risk [89].

A joint US-South Korean team estimated contents of different flavonoids in 15 commercial teas, including black and green teas and compared anticarcinogenic actions of individual tea compounds, such as nine green tea catechins, three black tea theaflavins, as well as extracts of the same tea leaves. Most tested compounds and all tea extracts inhibited growth of MCF-7 cell line. Other cell lines (colon, liver and prostate cell lines) were also inhibited. One of the conclusions from this study was that levels of the tea flavonoids did not directly correlate with anticancer activities. In addition, both green and black teas were found to possess similar anticancer potentials. In any case, consumers may benefit more by drinking both green and black teas [90].

Conflicting results obtained in epidemiological studies of black tea and coffee effects on development and progression of breast cancer may be due to shortcomings in precised control of hormone receptor status as well as in neglecting such factors as smoking, the dietary habits and frequency of daily intake. A Swedish group prospectively followed 61433 women who were cancer free in 1987–1990 and some of them later developed invasive breast cancer [91]. The results of this study indicate that black tea consumption may be positively associated with risk of ER+/PR+ tumors. The mentioned study revealed as well a non-significant correlation between increased coffee intake and breast cancer risk in ER+ group and inverse correlation in ER− group. More recent Swedish study provided strong evidence that coffee consumption decreased overall breast cancer risk of breast cancer. The risk decreased by 57 % (*P* = 0.0003) and 33 % (*P* = 0.034) For women who consumed more than five cups of coffee per day were the risks were 57 % (*P* = 0.0003) and 33 % (*P* = 0.034) in ER-negative and PR-negative groups disease, respectively [92]. No reduction in the incidence of breast cancer risk was found in ER-positive group.

## Wine as a Source of Potential Inhibitors of Breast Cancer

Grapes, one of the most popular fruits and the most widely cultivated throughout the world, contain large amounts of phytochemicals including anthocyanins and resveratrol (Fig. 1), which offer health benefits [93]. The beneficial health-related effects of phenolics in grapes are of importance to consumers, breeders and the grape industry.

Red wine is a rich source of polyphenolic components. The inhibitory effects of red wine polyphenolics on human breast cancer cells have been demonstrated earlier by many authors [94–98]. Grapes phenolics, flavonoids and resveratrol, from Pinot Noir, Cabernet Franc, Chardonnay, Catawba, Concord, Sheridan, Niagara and Riesling wines significantly inhibited the proliferation of Caco-2, HepG2 and MCF-7 human cancer cells [95]. Flavonoid fractions from red wine Merlot showed maximal inhibition of the growth of breast cancer cells, with relatively low cytotoxicity towards human mammary epithelial cells (HMEC) and human breast cancer (MCF-10A) cells [99], and MCF-7 breast cancer cells [100].

**Fig. 14:**
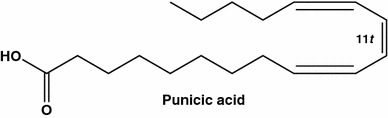
Rare natural acid, punicic acid (9*c*,11*t*,13*c*-18:3) from *Punica granatum*

Punicic acid (also known as trichosanic acid) is a long chain polyunsaturated fatty acid 9*c*,11*t*,13*c*-18:3 (Fig. 14) found in *Punica granatum* (pomegranate) seed oil (up to 80 %), extracts, and pomegranate red wine (Armenia). Pomegranate red wine contains up to 3 times more antioxidants than red wine [101]. One of the studies shows that pomegranate helps to slow down progression of prostate and breast cancers. Proliferation was inhibited 92 and 96 % for MDA-MB-231 and MDA-ERα7 cells, respectively compared to untreated cells by 40 µM punicic acid [102].

**Fig. 15:**
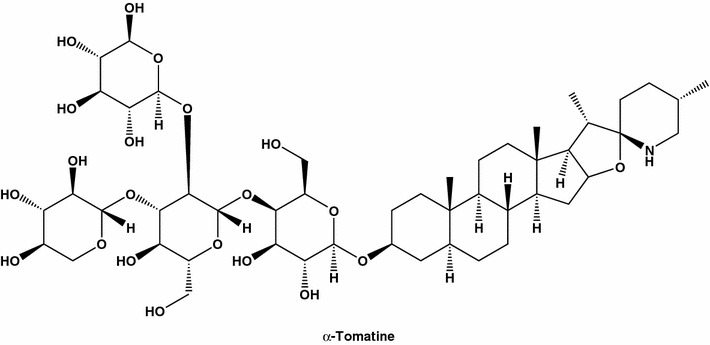
α-Tomatine also known as spiro[8H-naphth[2′,1′:4,5]indeno[2,1-b]furan-8,2′-piperidine], β-d-galactopyranoside derivative; spirosolane, or lycopersicin, is strong inhibitor of human breast (MDA-MB-231). Tomatine was isolated for the first time from extracts of crown gall-infected tomato plants

The traditional Chinese medicine wine usually manufactured from (wt%): *Venenum bufonis* (10–30), *Typhonium giganteum* (20–40), snake (10–30), and *Buthus martensii* (10–30) by slicing or pulverizing, soaking in hard liquor, and adding honey. This Chinese medicine wine can be used for treating breast cancer and pancreas cancer [103]. A health Chinese wine from tomato contains (wt%): wine yeast (1), tomato (25–30), white granulated sugar (3–4), and spice (0.5–1.0) [104]. The high-tomatine green tomato extracts strongly inhibited the following human cancer cell lines: breast (MCF-7), colon (HT-29), gastric (AGS), and hepatoma (liver) (HepG2), as well as normal human liver cells (Chang). The tomato glycoalkaloid α-tomatine (Fig. 15) was highly effective in inhibiting MCF-7 and all other tested cell lines [105].

## Concluding Remarks

From this hardly exhaustive review, one can make quite a simple conclusion [106–109]. The rejection of the conventional cancer treatment by the followers of traditional herbal medicine is reasonable due to the fact known by humans and every animal species: each plant and juice that could be consumed is safe. Quite often, however the traditionalists try to avoid another fact: the active plant ingredients in most cases represent toxins killing the cell, like taxol. Some of toxins derived from plants target numerous metabolic pathways. One of the examples is well known inhibitor of mitochondrial electron transport chain, rotenone [114–117].

In the nineteenth century, travelers in equatorial countries provided reports on the use of certain plants, belonging to the family *Fabaceae,* or *Papilionaceae*, for catching and killing fish. The fish poisoning plant ingredient was later identified as isoflavonoid. This “ichthyocide”, rotenone, has been widely used in solution as a pesticide and insecticide.

**Fig. 16:**
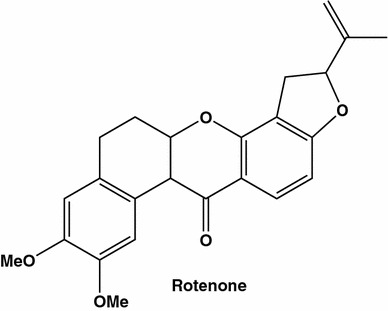
Rotenone is a naturally occurring compound extracted from the roots and leaves of leguminous [110–113]

Acting as a specific inhibitor of mitochondrial NADH dehydrogenase rotenone (Fig. 16) may increase the generation of ROS which represent an important proapoptotic factor. Indeed rotenone at micromolar concentrations was found to induce apoptosis in MCF-7 cells and this effect was attenuated in the presence of an antioxidant. The molecular mechanisms underlying the rotenone-induced effects were attributed to JNK and p38 MAPKs pathways activation and the inactivation of extracellular protein kinase 1/2 (ERK1/2). In addition, it increased level of apoptotic protein Bax while the level of antiapoptotic protein Bcl-2, was found to be decreased in the presence of rotenone in a time-dependent manner [107].

Traditional and modern approaches for prevention and treatment cancer may co-exist being complementary. Indeed, numerous tests on cancer cell survival and proliferation revealed strong additive or synergistic effects of combinations anti-cancer drug/extracts from a plant that may decrease unspecific toxicity of anticancer drugs. The synergistic action can not be reasonably explained without knowledge of the exact composition of presumably active ingredients in the extracts and juices. This action could be due to increased bioavailability or stability of the anticancer drugs, activation or inhibition of metabolic pathways modifying levels of pro- and anti-apoptotic proteins. In any case, in vitro experiments on cancer cell lines have to be confirmed by clinical studies.

Another probably less substantiated remark is that “safe” plant extracts would help cells to survive under conditions which progressively break intracellular machinery (one may mention free radical scavenging activities). On the other hand, the most efficient modern drugs kill cells, without discriminating normal and cancerous cells (to mention here free radical attacks). No matter that cancer cell, due to their extraordinary proliferative activity, are more vulnerable to DNA damaging or cell cycle blocking agents. A single healthy cell mutation can provoke its transformation into a malignant phenotype. In any case, combinations of drug treatment and traditional medicine have no sense and in some situation could be harmful for the patient.
